# The Speed of Neural Visual Motion Perception and Processing Determines the Visuomotor Reaction Time of Young Elite Table Tennis Athletes

**DOI:** 10.3389/fnbeh.2019.00165

**Published:** 2019-07-19

**Authors:** Thorben Hülsdünker, Martin Ostermann, Andreas Mierau

**Affiliations:** ^1^Department of Exercise and Sport Science, LUNEX International University of Health, Exercise and Sports, Differdange, Luxembourg; ^2^Fédération Luxemburgeoise de Tennis du Table, Route d’Arlon, Luxembourg; ^3^China Table Tennis College Europe, Route d’Arlon, Luxembourg; ^4^Institute of Movement and Neurosciences, German Sport University Cologne, Cologne, Germany

**Keywords:** sport, neuroscience, EEG, athlete, training, brain, vision, performance

## Abstract

**Purpose**: Recent research in adult badminton athletes has shown the visuomotor reaction time (VMRT) is strongly dependent on the speed of visual signal perception and processing in the brain’s visual motion system. However, it remains unclear if this relation can be confirmed for other visuomotor demanding disciplines as well as different age groups. This study aimed to validate previous findings in international elite youth table tennis players to shed light on the generalizability of neural performance determinants across different visuomotor demanding sports and age groups.

**Methods**: Thirty-seven young elite international table tennis players (18 male, 19 female, mean age: 13.5 years) from 23 nations participated in this study. Participants performed a visuomotor reaction task in response to visual motion stimuli presented at two different motion velocity conditions. Visuomotor performance was evaluated by measuring the electromyographic (EMG) onset as well as the VMRT. In addition, a 64-channel electroencephalography (EEG) system was used to investigate the stimulus and response-locked event-related potentials (ERPs) in the brain’s visual motion sensitive area MT as well as the pre- and supplementary motor cortex indicating the speed of cortical visual and motor information processing, respectively. Correlation and multiple regression analyses identified the neural processes determining visuomotor performance.

**Results**: The VMRT (232 vs. 258 ms, *P* < 0.001, *d* = −2.33) and EMG onset (181 vs. 206 ms, *P* < 0.001, *d* = −2.14) were accelerated in the fast motion velocity condition which was accompanied by an earlier stimulus-locked N2 (187 vs. 193 ms, *P* < 0.001, *d* = −0.80) and later response-locked N2-r (17 vs. −0.1 ms, *P* < 0.001, *d* = 1.04). The N2 and N2-r latencies were correlated with EMG onset and VMRT in both velocity conditions and explained between 80% and 90% of the variance in visuomotor reaction speed. Neural processes in BA6 did not differ between stimulus velocity conditions and did not contribute to the regression model.

**Conclusion**: The results validate our previous findings and support the importance of neural visual processes for the visuomotor reaction speed across different visuomotor demanding sports and age groups. This suggests the visual system might be a promising target for specific visual diagnostics and training interventions.

## Introduction

Table tennis is one of the fastest sports requiring athletes to perceive the ball and its trajectory within milliseconds to initiate a targeted motor response. Although table tennis players do not achieve the highest ball velocities when compared to other racquet sports such as tennis or badminton, the short distance between players requires extremely fast visuomotor reactions. Specifically, with ball velocities up to 10 ms^−1^ (Durey and Seydler, 1994) and a distance between players of only about 3 m, athletes have less than 500 ms to perform the movement.

Behavioral adaptations to these exceptionally high visuomotor demands in table tennis have previously been shown by Akpinar et al. (2012) in a coincidence-anticipation experiment where participants had to predict at what time an object arrived at a predefined target point. Over three different stimulus velocity conditions, table tennis players outperformed tennis and badminton athletes only during the fastest condition indicating particularly fast perception and processing of visual information. In addition to these findings on coincidence anticipation performance, visuomotor reaction experiments revealed faster reaction times in table tennis players when compared to non-athletes (Bhabhor et al., 2013) as well as experienced tennis players (Ak and Koçak, 2010; Can et al., 2014).

While these findings emphasize the crucial role of visuomotor reaction abilities in table tennis, previous research focused on behavioral data. However, it is now well established that especially the central nervous system determines the speed of visuomotor processes (Ando et al., 2001; Zwierko, 2008; Hülsdünker et al., 2018b). Therefore, it is essential to expand previous behavioral findings by determining the neurophysiological processes underlying faster visuomotor reactions in table tennis to provide more detailed information on the athletes’ visuomotor system and identify potential targets for specific training interventions.

In a recent series of studies, we determined the neural correlates of visuomotor reaction speed in elite badminton athletes and non-athletes. It was observed that the athletes’ superior reaction performance was accompanied by characteristic modulations in neural activity corresponding to visual and motor regions of the cerebral cortex. Specifically, following stimulus onset, athletes exhibited an earlier activation of the visual motion sensitive area MT as indicated by an earlier N2 potential (Hülsdünker et al., 2017, 2018a). The N2 can be observed around 170 ms following stimulus onset and is suggested to reflect the perception/processing of visual motion information (Kuba et al., 2007; Hülsdünker et al., 2017). Furthermore, athletes were characterized by a faster activation of the pre- and supplementary motor cortex [Brodmann area 6 (BA6)] as reflected by a lower latency of the BA6 negativity potential. The BA6 negativity potential occurs around 150 ms following stimulus onset which corresponds to stimulus-specific information processing (Schluter et al., 1999; Ledberg et al., 2007) and is interpreted as a process of transferring a visual signal into a motor command, referred to as visuomotor transformation (Hülsdünker et al., 2016, 2017). Early cortical potentials in MT and BA6 observable around 100 ms following stimulus onset reflected by the N1 and BA6 positivity, respectively did not differ between athletes and non-athletes thus likely reflecting an unspecific activation following visual stimulation (Ledberg et al., 2007; Hülsdünker et al., 2017).

In addition to these stimulus-locked components, athletes exhibited a later response-locked N2-r potential representing the time between electromyographic (EMG) onset and the N2 peak. A later N2-r indicates an earlier EMG onset relative to the N2 potential probably reflecting a more efficient integration of visual information (Hülsdünker et al., 2017). Importantly, these neurophysiological parameters not only differentiated athletes from non-athletes but explained about 66% of the variance in visuomotor reaction speed across groups. However, for elite athletes only the visual (N2, N2-r) but not motor processes (BA6 negativity) contributed to visuomotor reaction performance.

Although these findings emphasize the importance of specific neurophysiological and especially visual processes for the visuomotor reaction speed in badminton, it remains to be examined if the observed relations are generalizable to other visuomotor demanding sports. Since different sensory modalities and especially auditory information contribute to visuomotor performance in badminton (Yüksel and Tunç, 2018) and table tennis (Park et al., 2016), the relevance of visual information for the visuomotor reaction time (VMRT) may be different between the two sports, although visual information is obviously the most relevant source of sensory input. Further, our previous studies suggest the neurophysiological performance determinants for non-athletes and badminton players are different (Hülsdünker et al., 2017, 2018a) thus raising the question if this is particularly related to badminton or a feature of visuomotor demanding disciplines in general.

Alternatively, it would be plausible to assume that the comparable visuomotor demands of badminton and table tennis and particularly the importance of visuomotor reactions in both sports are associated with similar performance determining processes on the neural level. However, given the lack of result validation in science in general (Zwaan et al., 2017) and sport science in particular (Bernards et al., 2017; Halperin et al., 2018), our previous findings require validation to ensure that previously identified neurophysiological parameters are in fact present and performance determining in athletes participating in visuomotor demanding disciplines. This may be even more important in special populations such as elite athletes. Since the identification of athletes’ perceptual neurophysiological processes is a comparatively new field of research, a solid scientific basis is a prerequisite for future studies addressing neural functions for diagnostic and/or training purposes in elite sports.

However, in addition to the validation of recent findings in badminton players, this study will extend our previous approach and focus on young elite table tennis athletes. Given the higher plasticity of neural structures especially at younger ages (Paus et al., 2001; Bengtsson et al., 2005; Penhune, 2011), the identification of neural performance correlates in youth athletes is of special interest and could facilitate the development of specific training interventions, diagnostic tools or talent scouting approaches. This is particularly true since the results of Akpinar et al. (2012) as well as Ak and Koçak (2010) were obtained in young athletes between the age of 10–14 suggesting a substantial impact of visual perception and visuomotor reaction abilities already during early stages of table tennis training. Finally, while our previous research focused on a single visual motion speed (Hülsdünker et al., 2017, 2018a), this study will evaluate two motion velocity conditions thus allowing to draw conclusion not only about the generalizability for different disciplines but also motion speeds. Changes in visual motion speed have previously been shown to affect the latency of activation in the complex of area MT/MST (Kawakami et al., 2002; Maruyama et al., 2002; Hülsdünker et al., 2017) as well as behavioral reaction speed (Kawano et al., 1994; Genova et al., 2000; Kreegipuu and Allik, 2007) in both animals and humans. Specifically, a higher speed of the visual motion stimulus was associated with an earlier N2 component and faster visuomotor reactions. However, since Akpinar et al. (2012) reported differences between table tennis and badminton players only in the fast visual motion condition, there may be differences in the underlying neural processes determining visuomotor reaction performance at different visual motion speeds.

Based on the current state of research, this study aimed to extend previous findings in badminton athletes by identifying the neural determinants of VMRT in young elite table tennis players. To ensure comparability we used an identical visual motion stimulus as in our previous experiments. In addition to the VMRT, electroencephalography (EEG) and EMG were used to identify the stimulus-locked and response-locked cortical potentials of interest in visual and motor regions as well as the onset of muscular activity (EMG onset), respectively. Two stimulus velocity conditions were used to validate the results at slow and fast visual motion speeds.

Based on the abovementioned neurophysiological and behavioral studies, we expect the table tennis athletes to exhibit faster visuomotor reactions in the fast when compared to the slow stimulus velocity condition. This should be paralleled by faster neural activation in the visual system as reflected by a lower latency of the N2 and a higher latency of the N2-r potential. In addition to differences between conditions, we further expect the N2 and N2-r latencies to predict the visuomotor reaction speed as previously shown in badminton athletes. In contrast, motor processes in BA6 should play, if at all, only a minor role.

## Materials and Methods

### Subjects and Ethics

The study was conducted during two international table tennis training camps at the National Sports Institute [Institute National des Sports (INS)] in Luxembourg and in cooperation with the National Table Tennis Federation in Luxembourg (FLTT), the International Table Tennis Federation (ITTF), the European Table Tennis Union (ETTU), the China Table Tennis College Europe (CTTCE) as well as the German Sport University Cologne. All athletes participating in the training camps were nominated and invited by the ITTF or ETTU.

Thirty-seven highly experienced young table tennis athletes [18 male, 19 female, age: 13.5 years (±1.2), height: 162 cm (±9.3), weight: 51.1 kg (±10.5)] from 23 nations [Australia (1), Belgium (2), Croatia (1), Czech Republic (1), Egypt (3), France (2), Germany (4), Great Britain (2), Greece (1), Hongkong (2), Hungary (2), India (1), Italia (1), Lithuania (3), Mexico (1), Peru (1), Portugal (2), Russia (1), Singapore (1), Spain (1), Sweden (1), Thailand (1), USA (2)] participated in this study. All athletes participated in regular table tennis training for 7 years (±1) and had a weekly training load of 19 (±6) hours. Athletes compete at the highest level in their respective country and age group and regularly participate in national and international competitions and training camps. All participants confirmed having no history of neurological or psychiatric disorders and being free of injury at the test day including no limitation during their daily activities and/or training.

The experiment was an integral part of the training camps conducted by the ITTF, ETTU and CTTCE. All parents and athletes were provided detailed information about the study in advance of the training camp. Since all subjects were under the legal age, the parents gave their written informed consent prior to the study. Participants were informed about the experimental protocol on the test day and provided the opportunity to withdraw from the experiment at any point. The study was approved by the universities’ research ethics committee in accordance with the Declaration of Helsinki.

### Sample Selection and Size

This study was designed to validate our previous findings and especially confirm the relation between neural physiological processes and the VMRT in elite athletes. Therefore, we decided to not include a control group since our objective was to identify the neural correlates of visuomotor reaction performance in already highly trained athletes rather than evaluate differences between athletes and non-athletes. In badminton players (Hülsdünker et al., 2018a) the N2 and N2-r potentials were significantly related to EMG onset and VMRT with an *r*-value of at least 0.42. Based on these data, sample size calculations performed in G*Power 3.1.9.4 (Faul et al., 2009) as well as based on Hulley et al. (2013) resulted in a sample size of 39 and 42 participants respectively (two-tailed; α < 0.05; β = 0.8). With 37 participants included in this study, we were just below this threshold which can be justified by several reasons. First, the group in this study was clearly more homogeneous especially in terms of age and performance level when compared to the badminton players in our previous experiment. Therefore, we expect a stronger relation between neurophysiological and behavioral parameters and thus a greater effect size. Second, the sample size calculation was based on two-tailed significance tests. Given the directionality of N2 and N2-r correlations with EMG onset and VMRT confirmed in our previous studies (Hülsdünker et al., 2017, 2018a), it would also be reasonable to use one-tailed tests which would reduce the sample size to 31 (G*Power) or 34 (Hulley et al., 2013) participants. Third, there is only a limited number of young table tennis players that perform at a comparably high performance level and who are invited by the ETTU and ITTF to participate in international training camps. Increasing the number of participants would have been possible but only at the cost of losing sample homogeneity. Finally, our previous experiments with badminton athletes and only 36 participants still revealed significant relations between neurophysiological parameters (N2, N2-r) and the visuomotor reaction speed (EMG onset, VMRT). Therefore, considering the abovementioned arguments, we assume a sample size of 37 participants being appropriate for this study.

### Experimental Protocol

Participants were instructed to ensure at least 7 h of sleep prior to the test day and not to drink caffeinated drinks on the test day. An ophthalmologic test (Landolt test) was performed to ensure a visual acuity of at least 20/20. The Landolt test is the prescribed test to assess visual acuity according to the European norm EN ISO 8596 and is recommended for research settings (Grosvenor, 2007). Participants were placed 2 m away (test distance) from the Landolt visual acuity chart (eight circles) positioned at the subject’s eye level. Landolt rings were arranged according to the logarithm of the minimum angle of resolution (LogMAR). According to EN ISO 8596, the threshold to stop the test was 60% correct responses (five out of eight). Visual acuity values between 0.1 and 2 were assessed and subsequently convert to the Snellen index. The athlete’s playing hand was defined as the dominant hand for the visuomotor reaction tasks. Similar to our previous experiments (Hülsdünker et al., 2017, 2018a) as well as according to the recommendations of Kremlácek et al. (2004), the room was completely darkened during the experiment. The participant’s head was positioned on a stepless high adjustable chin-rest to ensure the eyes were level with the center of the screen. Participants were seated in a comfortable, high-adjustable chair, with their arms placed on the table. Earplugs were used to avoid any disturbance from surrounding noise. Further, athletes were instructed to not move during the experiment and specifically to avoid head movements. Before starting the experiment, all visual stimuli were presented during a practice session. The stimuli were presented binocularly while subjects had to keep their gaze at a fixation point presented at the center of the screen. Since previous research reported a stable gaze position when passively viewing optic flow pattern (Niemann et al., 1999), we abstained from using eye tracking to monitor the participant’s gaze position during the experiment. Overall the experiment lasted for about 20 min. The time between entering and leaving the laboratory was about 90 min.

The experiment consisted of two simple visuomotor reaction tasks. Athletes had to react in response to a visual motion stimulus presented at two different motion velocities. They were instructed to press a button on a response pad with the index finger of their dominant hand as fast as possible whenever they perceived a motion onset of the visual stimulus on the screen. To avoid temporal anticipation, the interstimulus intervals were randomized between 2 and 6 s. Each participant performed 80 trials for each motion velocity condition, subdivided into four blocks of 20 stimuli. Four sequences were performed, each containing one block of the two velocity conditions, respectively. The order of blocks within each sequence was randomized. Within each block of 20 trials there was a pause of 15 s after 10 reactions. Pauses between two blocks and between two sequences were 20 and 30 s, respectively.

### Visual Stimuli

**Figure 1:**
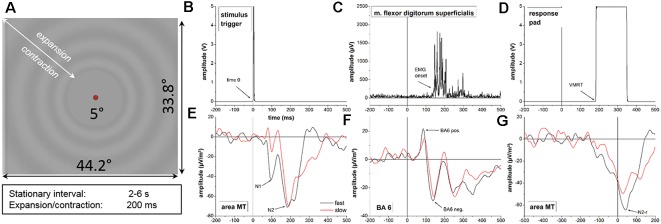
**(A)** Radial visual motion onset stimulus used in this experiment. **(B–G)** Representative data of a single subject during the visuomotor reaction experiment. Data values for the stimulus trigger **(B)**, electromyographic (EMG) onset **(C)** and the response pad **(D)** are displayed for a single trial of the fast motion onset condition. Time-locked neurophysiological activity in area MT **(E)** and BA6 **(F)** as well as response-locked data in are MT **(F)** represent averaged time courses of cortical activity across single trials for the slow (red line) and fast (black line) motion onset condition. Behavioral and neurophysiological parameters investigated in the experiment are highlighted with arrows. Note the different scaling on the x-axis for the response-locked activity in area MT **(G)** due to different segmentation. Time 0 = stimulus onset; VMRT = visuomotor reaction time (button press); BA6 pos. = BA6 positivity; BA6 neg. = BA6 negativity.

Visual stimuli were programmed with the CRS toolbox (Cambridge Research Systems, Rochester, UK) implemented in Matlab (The Mathworks, Natick, MA, USA) and presented using a ViSaGe MKII stimulus generator (Cambridge Research Systems), on a 22-inch cathode ray tube (CRT) Monitor (HP1230 Color CRT, Hewlett Packard Enterprise, San Jose, CA, USA) with a refresh rate of 120 Hz. The visual stimuli and stimulation settings were identical to our previous motion onset experiments with badminton players (Hülsdünker et al., 2017, 2018a). Specifically, the viewing distance was set to 500 mm and a radial motion onset stimulus with a mean luminance of 17 cd∙m^−2^ was used for visual stimulation. According to the recommendations of Kremlácek et al. (2004), the temporal stimulus frequency was kept constant by decreasing the spatial frequency towards the periphery. The visual stimulus subtended a visual field of 44.2° × 33.8° and contained a stationary gray circle of 5° at the middle of the screen (luminance = 17 cd∙m^−2^) with a red fixation point at its center (luminance = 17 cd∙m^−2^). The stimulus luminance was sinusoidally modulated with a Michelson contrast of 10% to achieve a more specific activation of the magnocellular system. The luminance values for all pixels were updated at a rate of 120 Hz (frame rate) according to pre-defined luminance values stored in look-up-tables (LUTs). By cycling through these LUTs, the visual stimuli appeared to move on the screen. By either expanding or contracting the motion adaptation effect across trials was reduced. Since the stimulus was designed to keep the temporal frequency constant, its velocity is described in Hertz (Hz). The radial motion onset visual stimulus used in this experiment is presented in Figure 1. For this experiment, a slow motion condition at a stimulus velocity of 5 Hz and a high velocity condition of 20 Hz were used. The 5 Hz condition was identical to our previous experiments while the 20 Hz condition was used to validate the results also for higher stimulation frequencies (higher motion velocity). For both conditions, the stimulus moved for 200 ms while the interstimulus interval was randomly varied between 2 and 6 s. The mean luminance and Michelson contrast were checked using a ColorCallII colorimeter (Cambridge Research Systems). During the experiment all recording systems were synchronized by electrical trigger pulses frame-synchronously generated by the ViSaGe MKII stimulus generator.

### Data Recording

EEG data were recorded using a 64-channel actiChamp amplifier (Brain Products GmbH, Gilching, Germany). Sixty-three active electrodes were equally distributed over both hemispheres according to the 10:10 system (Jurcak et al., 2007) while one electrode was used to record electrooculographic signals. The ground and reference electrodes were placed on AFz and FCz, respectively. Electrode impedances were kept below 15 kΩ and data were recorded with an online low-pass filter of 280 Hz. For EMG measurements, one DE-2.1 double differential surface EMG sensor (Bagnoli; Delsys, Natick, MA, USA) with a contact spacing of 10 mm and an input impedance of >10^15^ kΩ was placed on the flexor digitorum superficialis muscle of the dominant arm. EMG data were online band-pass filtered by the recording system between 20 and 450 Hz. The VMRT was determined by a button press with the index finger of the dominant hand on a Cedrus RB-530 response pad (Cedrus, San Pedro, CA, USA). All recording devices were sampled with a frequency of 1000 Hz.

### Data Analysis

#### EEG Data Analysis

EEG data were analyzed using the Brain Vision Analyzer 2 software (Brain Products GmbH), as well as scripts of the EEGlab toolbox (Delorme and Makeig, 2004) implemented in Matlab.

EEG data were first band-pass filtered between 0.3 and 35 Hz, segmented into epochs of 1500 ms length (−500 to 1000 ms relative to stimulus onset) and the baseline (−500 to 0 ms) was subtracted. Based on an ocular correction independent component analysis (ICA) algorithm implemented in the Brain Vision Analyzer 2 software, eye blinks in an interval between −500 and 200 ms were identified. Segments contaminated by artifacts within that interval were excluded due to an expected impairment of task-related visual perception/processing processes. An additional semiautomatic artifact rejection procedure (interval: −500 to 500 ms) excluded segments with voltage steps >50 μV or a positive or negative amplitude exceeding ±150 μV. All segments were also visually checked for artifacts. On average, 3.7 (± 2.8) and 4.1 (± 2.6) of the segments were excluded for the fast and slow motion onset condition, respectively. Noisy or artifactual channels as identified by >25% artifactual segments were deleted and interpolated after ICA-based artifact correction. In EEGlab (Delorme and Makeig, 2004), a runica algorithm was applied for ICA decomposition. Components reflecting artifacts (especially muscular activity or eye blinks after 200 ms following the visual stimulation) were excluded. After ICA back transform, the current source density (CSD) was calculated (number of splines = 4, maximal degrees legendre = 10, Lambda = 1e^−5^) and all segments were averaged.

#### EMG Data Analysis

EMG data were first low-pass filtered, segmented similar to the EEG data (−500 to 1000 ms) and the baseline activity (−500 to 0 ms) was subtracted. According to the procedure of Hodges and Bui (1996), the EMG onset was defined when the 50-Hz low-pass filtered and smoothed (25 ms moving average) signal exceeded three standard deviations from baseline (−500 to 0 ms). For each trial, the EMG onset was visually checked and corrected if necessary. Trials with an EMG onset <100 ms following stimulus onset were excluded.

#### Identification of Visuomotor Reaction Time (VMRT) and Motor Time

The VMRT was defined as the time between stimulus presentation (motion onset) and button press (movement execution). The motor time reflected the time between EMG onset and VMRT. Trials with a VMRT <100 or >500 ms were excluded. Further, trials with a reaction time that exceeded the average VMRT by ±3 standard deviations were deleted. In the same vein, we excluded trials indicating a motor time ±3 standard deviations of the average motor time (indicating erroneous EMG onset definition).

After all artifact and segment rejection procedures, 73 (±4) and 72 (±5) segments corresponding to 91% and 90% of the 80 reaction trials were included in data analysis for the fast and slow motion onset condition, respectively.

#### Definition of Cortical Potentials

To identify the and N1 and N2 ERPs in the visual motion sensitive area MT, we calculated the average activity of electrode positions PO7, P7, P5, PO8, P8 and P6. In our previous experiment using an identical visual motion stimulus (Hülsdünker et al., 2017), these electrodes have been identified to best represent area MT according to the cortical projections of EEG electrodes in the 10:10 electrode system (Koessler et al., 2009). Due to variations in MT localization across individuals, electrode positions, TP9, TP7, T7, TP10, TP8, P8 were considered for participants with a more anterior localization of area MT (Hülsdünker et al., 2017). We used a similar approach to identify the positivity and negativity potentials in BA6 by averaging electrode positions FC1, FCz and FC2 that represented the cytoarchitectonic region of BA6 in all subjects according to the study of Koessler et al. (2009). To further account for interindividual differences we also included electrode positions F1, F2, Fz, FC3, FC5, FC6, FC4, that were all found to correspond to BA6 in >50% of the participants (Koessler et al., 2009). However, activity in these channels was weighted by the factors 0.61, 0.69, 0.82, 0.75, 0.63, 0.57 and 0.82, respectively reflecting their probability of contributing to BA6 (Koessler et al., 2009).

Based on the averaged cortical activity across all segments in area MT, the N1 and N2 potentials were defined as the maximal negative peaks within the interval between 50 and 150 ms and 100–300 ms, respectively. The BA6 positive and BA6 negativity potentials reflected the maximal positive peak between 50 and 150 ms as well as the maximal negative peak between 100 and 300 ms, respectively. All peaks were checked visually and adjusted if necessary.

In addition to the stimulus-locked components, the N2-r potential in area MT was identified based on response-locked data. To this end, all single trials were re-segmented into epochs between −500 and 200 ms relative to the EMG onset. Based on the average response-locked cortical activity in area MT across epochs, the N2-r was defined as the maximal negative peak between −50 and 50 ms relative to EMG onset. Similar to response-locked ERPs, all peaks were visually checked.

To evaluate the underlying neural sources of the visual and motor ERPs of interest (N2, N2-r and BA6 negativity), we conducted an inverse localization analysis within a 20 ms pre-peak window around the N2, N2-r and BA6 negativity potentials using the LORETA localization module implemented in the Brain Vision Analyzer software. The LORETA model is based on 2394 voxels with a resolution of 7 mm in a 3-spherical volume conductor head model fitted to the MNI-305 brain template co-registered to the Talairach brain atlas.

### Statistical Analysis

Statistical analyses were performed using the Statistica 7.1 software package (StatSoft, Tulsa, OK, USA).

Paired *t*-tests were performed to compare muscular (EMG onset, motor time), behavioral (VMRT) and neurophysiological (N1, N2, BA6 positivity, BA6 negativity, N2-r) parameters between the fast and the slow motion onset condition. Amplitude and latency values were compared for all neurophysiological parameters, while only latency was considered for EMG onset, motor time and VMRT. Kolmogorov-Smirnoff tests confirmed normal distribution of all parameters as well as of difference scores between conditions. To account for multiple *t*-test comparisons, false-discovery rate (FDR) correction was applied.

To identify direct relations between neurophysiological activity and visuomotor reaction performance, all ERP amplitude and latency parameters were correlated to EMG onset and VMRT using Pearson correlation coefficients. In addition, stepwise forward multiple regression models were calculated to predict EMG onset and VMRT by ERP latency and/or amplitude. To reduce the number of predictors, only parameters that were significantly correlated with EMG onset and/or VMRT were considered for the regression analyses.

To check for multicollinearity, we calculated tolerance as well as the variance inflation factor (VIF). Parameters indicating tolerance <0.4 (VIF >2.5) were excluded from regression analyses. In addition, variables without normal distribution of residuals were not considered. Breusch-Pagan and Durbin-Watson tests checked for homoskedasticity and autocorrelation, respectively.

Effect sizes were defined based on the thresholds suggested by Cohen (1988) and considered small (*d* = 0.2; *r* = 0.1), medium (*d* = 0.5; *r* = 0.3) or large (*d* = 0.8, *r* = 0.5). Significance levels were defined as follows: *P < 0.05, **P < 0.01 and ***P < 0.001.

## Results

Representative behavioral, muscular and neurophysiological data from a single subject are displayed in Figure 1.

### Fast vs. Slow Motion Onset Condition

Table 1 presents the mean values for all parameters in the fast and slow motion onset condition. For the muscular and behavioral data, *t*-tests revealed a significantly earlier EMG onset (*t*_(36)_ = −13.03, *P* < 0.001, *d* = −2.14) as well as a faster VMRT (*t*_(36)_ = −14.19, *P* < 0.001, *d* = −2.33) in the fast when compared to the slow motion onset velocity condition. In contrast, there was no difference for the motor time (*t*_(36)_ = −0.96, *P* = 1, *d* = −0.15).

**Table 1:** Behavioral and neurophysiological parameters obtained during the slow and the fast visual motion onset condition averaged across participants.

Parameter		Unit	Condition	
			Slow motion onset	Fast motion onset
Behavioral	EMG onset	ms	206.4 (±9.9)	181.2 (±8.4)***
	VMRT	ms	258.4 (±10.1)	232.9 (±9.7)***
	Motor time	ms	51.9 (±3.1)	51.3 (±3.3)
Neurophysio logical
Visual	N1 latency	ms	107.3 (±3.1)	103.4 (±3.5)*
	N1 amplitude	μV/m^2^	−15.5 (±3.9)	−27.4 (±6.0)***
	N2 latency	ms	193.4 (±6.0)	187.1 (±6.0)***
	N2 amplitude	μV/m^2^	−54.9 (±8.4)	−67.0 (±10.1)***
	N2-r latency	ms	−0.1 (±7.6)	17.2 (±7.2)***
	N2-r amplitude	μV/m^2^	−43.4 (±7.4)	−53.3 (±9.0)***
Motor	BA6 positivity latency	ms	88.9 (±3.9)	87.7 (±4.9)
	BA6 positivity amplitude	μV/m^2^	5.3 (±1.9)	6.3 (±1.9)
	BA6 negativity latency	ms	161.8 (±6.4)	164.5 (±8.1)
	BA6 negativity amplitude	μV/m^2^	−13.7 (±4.2)	−17.5 (±4.9)

Data values reflect mean (±95% confidence interval). *p < 0.05; ***p < 0.001 difference between the slow and fast motion onset condition.

In the visual motion sensitive area MT, the fast motion onset condition was characterized by an earlier N2 potential (*t*_(36)_ = −4.91, *P* < 0.001, *d* = −0.80) following stimulus presentation as well as a later N2-r potential relative to EMG onset (*t*_(36)_ = 6.34, *P* < 0.001, *d* = 1.04) when compared to the slow motion onset condition. A difference between conditions was also observed for the N1 latency (*t*_(36)_ = −2.80, *P* = 0.049, *d* = −0.46) indicating a lower N1 latency in the fast when compared to the slow motion onset condition.

The significant latency differences between the two velocity conditions were highly consistent across participants. Specifically, all athletes exhibited an earlier EMG onset and a faster VMRT in the fast when compared to the slow velocity condition. A lower N2 in the fast condition was observed in 27 out of 37 participants (73%). Eight athletes (22%) did not indicate a change in N2 latency and a small increase was observed in two subjects (5%). For the N2-r, latency values were higher in the fast when compared to the slow condition in 32 athletes (87%) with no change in 3 (8%) and a decrease in two (5%) participants.

In addition to latency differences, the fast motion onset condition exhibited a greater peak negativity of the N1 (*t*_(36)_ = −6.10, *P* < 0.001, *d* = −1.00), N2 (*t*_(36)_ = −5.01, *P* < 0.001, *d* = −0.82) and N2-r (*t*_(36)_ = −5.34, *P* < 0.001, *d* = −0.83) potentials.

**Figure 2:**
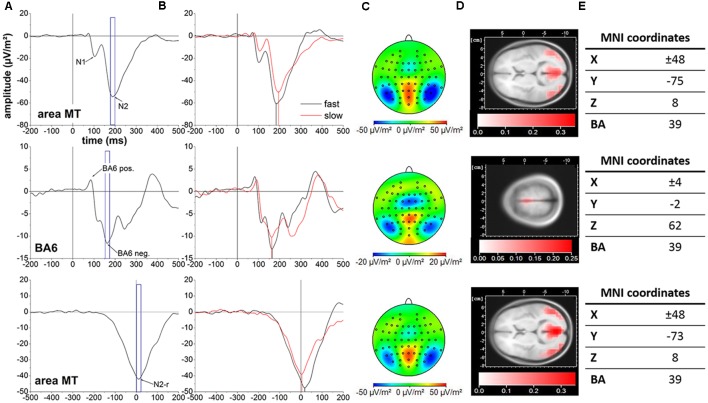
Cortical activity averaged across all 37 subjects. Top row: stimulus-locked data in area MT. Middle row: stimulus-locked data in BA6. Bottom row: response-locked date in area MT. The event-related potentials (ERPs) of interest are indicated with arrows. Blue frames represent the 20 ms peri-peak window centered at the N2 **(A)**, BA6 negativity **(B)** and N2-r **(C)** potentials forming the basis for cortical mappings and LORETA localization in **(C)** and **(D)**, respectively. **(B)** Cortical activity for the slow (red line) and fast (black line) motion onset condition. Vertical black and red lines illustrate the temporal projection of the N2, BA6 negativity and N2-r potentials. **(C)** Cortical mappings reflecting the N2, BA6 negativity and N2-r potentials based on the 20 ms peri-peak windows in **(A)**. **(D)** LORETA localization of the N2, BA6 negativity and N2-r potentials based on the 20 ms peri-peak windows in **(A)**. **(E)** LORETA based MNI coordinates and corresponding Brodmann areas (BA) representing the location of maximal cortical activity associated with the N2, BA6 negativity and N2-r potentials. Please note the differences in scaling between cortical activity in area MT and BA6 for cortical mappings and LORETA localization.

For the pre- and supplementary motor region (BA6), both the BA6 positivity and BA6 negativity did not yield a difference in latency between conditions (BA6 positivity: *t*_(36)_ = −0.47, *P* = 0.639, *d* = −0.08; BA6 negativity: *t*_(36)_ = 0.81, *P* = 0.840, *d* = 0.13). Further, also the BA6 positivity and BA6 negativity amplitudes were similar between conditions (BA6 positivity: *t*_(36)_ = 1.31, *P* = 0.793, *d* = 0.22; BA6 negativity: *t*_(36)_ = −2.60, *P* = 0.066, *d* = −0.43). Time courses of cortical activity in area MT and BA6 across subjects for the slow and fast motion onset conditions as well as corresponding cortical mappings and LORETA localization results are displayed in Figure 2.

### Neurophysiological Data Predicting Visuomotor Performance

For both the fast and slow visual motion onset conditions, the N2 correlated with the EMG onset (fast: *r*_(37)_ = 0.59, *P* < 0.001; slow: *r*_(37)_ = 0.58, *P* < 0.001) and the VMRT (fast: *r*_(37)_ = 0.55, *P* < 0.001; slow: *r*_(37)_ = 0.60, *p* < 0.001). A similar pattern of results was also observed for the N2-r potential likewise indicating a direct relation to EMG onset (fast: *r*_(37)_ = −0.82, *P* < 0.001; slow: *r*_(37)_ = −0.87, *P* < 0.001) and VMRT (fast: *r*_(37)_ = −0.80, *P* < 0.001; slow: *r*_(37)_ = −0.87, *P* < 0.001). A further correlation was observed for the N1 latency although this was restricted to the slow motion onset condition (EMG onset: *r*_(37)_ = 0.45, *P* = 0.005; VMRT: *r*_(37)_ = 0.38, *P* = 0.021). In contrast, EMG onset and VMRT were not correlated to the BA6 positivity (EMG onset: slow: *r*_(37)_ = 0.31, *P* = 0.063; fast: *r*_(37)_ = −0.002, *P* = 0.991: VMRT: slow: *r*_(37)_ = 0.24, *P* = 0.145; fast: *r*_(37)_ = 0.04, *P* = 0.816) or BA6 negativity (EMG onset: slow: *r*_(37)_ = 0.15, *P* = 0.386; fast: *r*_(37)_ = -0.10, *P* = 0.556; VMRT: slow: *r*_(37)_ = 0.17, *P* = 0.303; fast: *r*_(37)_ = −0.31, *P* = 0.855) latency in both the slow and fast visual motion onset condition.

**Figure 3:**
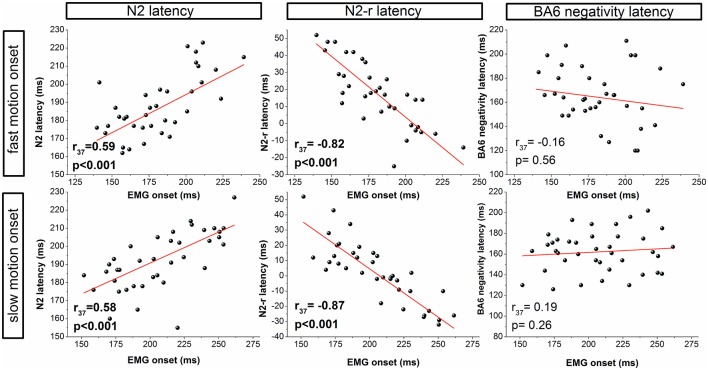
Correlation analyses for the fast (top row) and slow (bottom row) visual motion onset condition. Correlations between EMG onset and the latency of ERPs are presented for visual (N2, N2-r) and motor (BA6 negativity) components.

For both motion onset conditions, the N2 and N2-r latencies were included as independent variables while the N1 latency was added only for the slow condition. In the fast motion onset condition, the regression model corrected for the number of input variables revealed the visual parameters explained about 90% of the variance for EMG onset (*F*_(2,34)_ = 161.42, *r*^2^_corrected_ = 0.90, *P* < 0.001) and 83% for VMRT (*F*_(2,34)_ = 88.66, *r*^2^_corrected_ = 0.83, *P* < 0.001). For the slow motion condition, the N1 latency component was excluded as a predictor by the multiple regression algorithm for both EMG onset and VMRT. The remaining N2 and N2-r components predicted 79% of the variance for EMG onset (*F*_(2,14)_ = 46.77, *r*^2^_corrected_ = 0.79, *P* < 0.001) and 81% for VMRT (*F*_(1,15)_ = 76.77, *r*^2^_corrected_ = 0.81, *P* < 0.001). Control analyses confirmed the absence of multicollinearity, non-normal distribution of residuals, heteroscedasticity and autocorrelation for all variables and regression models. Figure 3 presents the correlation and regression results.

### LORETA Localization (BA6 Negativity, N2, N2-r)

Cortical locations of the N2, N2-r and BA6 negativity potentials associated with the fast and slow motion onset conditions are displayed in Figure 2E.

## Discussion

While visuomotor reactions are well established to play a performance determining role in table tennis, this study is the first to identify the neural processes associated with superior visuomotor reaction performance in international young elite table tennis players. Faster visuomotor reactions were associated with an acceleration of visual processes in the motion sensitive area MT while motor processes were not related to the reaction speed. This applied to both, the difference between the two motion onset velocity conditions as well as interindividual performance discrepancies between athletes. The findings support our previous studies in badminton players and emphasize the generalizability of performance determining neurophysiological processes across different visuomotor demanding disciplines. When considering the limited number of studies addressing neural visual processes in elite athletes, the results are essential to validate the performance-determining role of visual perception and processing speed for visuomotor reactions. Further, this study expands our results in adult athletes by establishing a substantial relation between neurophysiology and reaction abilities already for young athletes. Given the higher neural plasticity at a younger age, this information highlights the potential of neurophysiological and especially visual processes for diagnostic and training purposes in visuomotor demanding sports.

### LORETA Localization of Cortical Potentials

The cortical localization of the N2 and N2-r potentials in both the fast and slow visual motion conditions is well in line with previous human and animal studies on the location of the motion sensitive area MT (Dumoulin et al., 2000; Maruyama et al., 2002). Further, the MT coordinates obtained in this experiment match previous results in badminton players (Hülsdünker et al., 2017). Therefore, we suggest the N2 and N2-r potentials originate from the human motion sensitive area MT, a key region for visual motion perception/processing (Born and Bradley, 2005). Similar to our previous research, the BA6 positivity and negativity potentials were located in the pre- and supplementary motor cortex (BA6).

### Fast vs. Slow Motion Onset Condition

In line with our first hypothesis, this study confirmed significantly faster visuomotor reactions accompanied by an earlier activation of the visual motion specific area MT in the fast when compared to the slow-motion velocity condition.

#### Behavioral Data

The results on EMG onset and VMRT indicate a faster VMRT in response to fast when compared to slow visual motion onset stimuli. These findings are in line with numerous previous studies using motion onset as well as motion direction change paradigms likewise indicating an acceleration of reaction time with increasing stimulus speed (Genova et al., 2000; Kreegipuu and Allik, 2007). When considering the identical motor time for both stimulus velocity conditions this study confirms previous research suggesting the VMRT is primarily determined by the signal processing speed in the central nervous system (Ando et al., 2001; Zwierko, 2008; Hülsdünker et al., 2017).

#### Neurophysiological Data

Cortical ERPs were identifiable in visual and motor regions. Comparable to our previous study in elite badminton players (Hülsdünker et al., 2017), especially visual processes contributed to differences in reaction time. In contrast, motor processes only played, if at all, a minor role.

#### The Visual Motion Sensitive Area MT

Activation in the visual motion sensitive area MT was characterized by an early negative potential with an onset around 100 ms after stimulus presentation. This N1 potential did not differ between the two motion onset velocity conditions suggesting an unspecific early activity independent from the visual stimulus characteristics (i.e., motion velocity) instead of stimulus-specific visual processing. That interpretation is in line with animal experiments in monkeys likewise suggesting an unspecific early visual activation that is not related to visual stimulus processing. Specifically, Ledberg et al. (2007) investigated activation in the monkey visual cortex corresponding to human area MT in a visuomotor Go-NoGo task. While an initial peak of activation was observed around 100 ms that well matches the N1 potential in this study, differences between stimulus conditions occurred only after about 150 ms following the stimulus. In the same line of reasoning, a disruption of area MT function by transcranial magnetic stimulation (TMS) in humans has been shown to affect performance in a motion perception task only between 130 and 150 ms following motion onset (Sack et al., 2006; Stevens et al., 2009). This combined pattern of results suggests the N1 to reflect an early but unspecific activation of area MT, followed by stimulus-specific processing.

The reported latencies of task-related visual processing starting around 130–150 ms correspond well to the onset of the N2 potential observed in area MT. The N2 has previously been suggested to reflect the cortical correlate of visual motion perception/processing (Kuba et al., 2007). In this study, faster visuomotor reactions in the fast motion velocity condition were associated with a lower N2 latency indicating faster visual motion perception with increasing stimulus speed. These findings support previous research suggesting an interrelation between visual motion speed and MT activation latency as well as MT activation latency and visuomotor reaction performance. Specifically, an increase in visual motion speed has been shown to reduce the MT activation latency (Kawakami et al., 2002) that in turn is associated with faster visuomotor reactions (Kawano et al., 1994; Kawakami et al., 2002). Moreover, the findings correspond to our recent experiments with badminton players were a lower N2 latency contributed to faster reactions in athletes when compared to non-athletes (Hülsdünker et al., 2017). Therefore, this study adds further support to the assumption that a higher visual motion speed accelerates stimulus-specific information processing in area MT (N2) that in turn allows faster visuomotor reactions (EMG onset/VMRT).

In addition to the two stimulus-locked parameters (N1 and N2), this study also examined the response-locked N2-r component in area MT. The N2 and N2-r reflect the same potential which is indicated by high correlations between amplitudes (fast: *r*_(37)_ = 0.95; slow: *r*_(37)_ = 0.94) and is in accordance with previous findings in badminton athletes and non-athletes (Hülsdünker et al., 2017). The evoked N2-r potential suggests visual perception/processing is not only time-locked to stimulus presentation but also to the motor response as reflected by the EMG onset. Similar results have previously been reported during ocular following responses in monkeys (Kawano et al., 1994; Hietanen et al., 2017) suggesting movement execution is temporally tied to the preceding perception and processing of visual information. In this study, the N2-r occurred later relative to EMG onset in the fast condition suggesting faster processing of visual motion information at higher stimulus velocities. However, interestingly, the N2-r was observed around EMG onset and especially in the fast visual motion condition, even before muscle activation. While this may contradict mental chronometry, the results add further support to the assumption, that visual motion perception/processing is not necessarily reflected by the peak activation but instead an activation threshold. Specifically, Stevens et al. (2009) suggested a successive integration of visual information over time. Based on this model, the faster motion onset stimulus providing more visual motion information (rate of luminance change) per time should reach this threshold earlier and allow a faster initiation of the motor response. Consequently, the EMG onset in response to fast visual motion stimuli occurs earlier in relation to the N2-r potential when compared to the slow-motion condition.

In addition to changes in latency, the faster motion onset condition was characterized by an increase in the N1, N2 and N2-r amplitude. This has previously been reported in an MEG study by Kawakami et al. (2002) and may be attributable to an increasing number of activated neurons as observed in single-cell recordings (Kawano et al., 1994) resulting from a greater stimulus intensity (motion speed).

In sum, the results on the N2 and N2-r potentials in area MT suggest both, a faster visual motion signal processing as well as a greater neural activation for the fast when compared to the slow stimulus velocity condition that is likely attributable to the higher motion velocity of the visual stimulus.

#### The Pre- and Supplementary Motor Region BA6

The early BA6 positivity potential was observed around 100 ms after stimulus onset that well matches the N1 timing in area MT. Further, like the N1, there were no differences in BA6 positivity amplitude or latency between the two motion velocity conditions. Again, these findings are well in accordance with the results of Ledberg et al. (2007) reporting a widespread cortical activation in response to visual stimulation that did not only include visual but also cortical motor regions. The authors observed that comparable to the N1, also the early motor activation was not associated with stimulus-specific processing. These results are also supported by a TMS study of Schluter et al. (1999) indicating a disruption of the pre-motor cortex in BA6 around 100 ms did not affect the VMRT. Based on these findings we suggest the BA6 positivity to reflect an unspecific activation of motor regions in response to visual motion stimulation that is not associated with stimulus processing and thus not sensitive to differences in visual stimulus velocity.

The later BA6 negativity potential was identified around 160 ms following stimulus onset and has previously been interpreted as a process of visuomotor transformation (Hülsdünker et al., 2016, 2017). In fact, Ledberg et al. (2007) suggested stimulus-specific processing in pre- and supplementary motor regions starting around 150 ms following visual stimulation. A similar period has also been observed by Schluter et al. (1999) who reported an impairment of visuomotor reactions when applying TMS to the premotor cortex between 140 and 180 ms after visual stimulus onset. However, although these findings suggest the functional significance of BA6 within a time interval that corresponds to the BA6 negativity peak, we observed no differences between the two velocity conditions for both the BA6 negativity latency and amplitude. This adds further support to the hypothesis suggesting the speed of motor processes only plays a minor role for simple visuomotor reactions. The underlying reason may be associated with the visuomotor task characteristics. Specifically, visual and motor processes in the cortex seem to act at least partly independent from each other. Since this experiment modulated the visual stimulus but not the motor response, which was a button press with the index finger for both stimulus velocity conditions, there may be no change in visuomotor transformation and thus BA6 activation. Accordingly, while the N2 and N2-r components change with variations in visual stimulus characteristics (i.e., speed), the BA6 negativity remained constant.

### Neurophysiological Processes Predicting Visuomotor Performance

#### The Visual Motion Sensitive Area MT

While modulations in cortical activity associated with modulations in visual motion velocity confirmed our first hypothesis, we further observed a direct relation between neural processes in the visual system and visuomotor reaction performance. This applied to both, the slow and fast motion condition where the reaction time was predicted by the N2 latency which is in accordance with the second hypothesis of this study. Further these findings also support previous research likewise suggesting an interrelation between visual motion speed and MT activation latency as well as MT activation latency and visuomotor reaction performance. Specifically, an increase in visual motion speed has been shown to reduce the MT and MST activation latencies that in turn are associated with an accelerated motor response initiation (Kawano et al., 1994; Kawakami et al., 2002). Moreover, the findings correspond to our recent experiments with badminton players were a lower N2 latency contributed to faster reactions in athletes when compared to non-athletes and determined the visuomotor reaction speed in already highly experienced badminton players (Hülsdünker et al., 2017, 2018a). Therefore, this study adds further support to the assumption that a faster visual signal perception/processing in area MT as reflected by the N2 latency accelerates the athlete’s visuomotor reaction speed.

In addition to the stimulus-locked N2 potential also the response-locked N2-r VEP was directly related to behavioral reaction performance. Specifically, the N2-r was the best predictor for visuomotor reaction performance and was negatively correlated to EMG onset and VMRT. In other words, athletes with a faster reaction time were characterized by a later occurrence of the N2 peak relative to the onset of muscular activation. Similar results have previously been observed in badminton athletes (Hülsdünker et al., 2018a). With regard to the abovementioned threshold model for visual motion integration these findings suggest that athletes with faster visuomotor reactions may achieve a more efficient integration of motion information in area MT that allows an earlier initiation of the motor response.

The combined pattern of results in the visual motion sensitive area MT confirm our previous studies with badminton players and emphasize the performance-determining role of visual perception and processing speed for visuomotor reactions across disciplines and age groups. The strong relation between neurophysiology and behavioral performance observed in the young table tennis players is of particular interest given the higher neural plasticity of gray and white matter at younger ages (Paus et al., 2001; Bengtsson et al., 2005; Penhune, 2011). For instance, there is a substantial increase in myelination during development that is not fully laid until the age of 20–30 years (Paus et al., 2001; Fields, 2008). Importantly, myelination starts at the back of the head thus including visual regions at relatively early stages of development (Fields, 2008). In line with this the N2 latency substantially decreases by about 74 ms between the age of 8 and 18 years (Langrová et al., 2006; Kuba et al., 2007). Since neural plasticity of white and gray matter can be modulated by experience, as indicated in animal (Sirevaag and Greenough, 1987) and human (Scholz et al., 2009) experiments, training interventions specifically addressing visual functions may be particularly promising for young athletes. In some previous studies, stroboscopic training has been shown to improve visual motion sensitivity (Appelbaum et al., 2011) and sport-specific visuomotor reaction performance (Hülsdünker et al., 2019), both processes that crucially involve area MT.

#### The Pre- and Supplementary Motor Region BA6

For the pre- and supplementary motor region, both the BA6 positivity and negativity did not correlate to the visuomotor reaction speed or contribute to the regression model. Accordingly, when compared to visual processes, the visuomotor reaction speed of table tennis players seem to be largely independent of signal processing speed in BA6. While this may be surprising with regard to previous group comparisons (Hülsdünker et al., 2017), it is in line with our analysis in badminton players (Hülsdünker et al., 2018a). Specifically, although the BA6 negativity latency differentiated athletes from non-athletes, there was no relation to the visuomotor reaction speed within the group of already highly experienced badminton players. Since we focused on a homogeneous group of young elite table tennis players this likely explains the missing relation between BA6 negativity and visuomotor reaction speed. In sum, the combined results of badminton and table tennis athletes indicate that on a high performance level, visual processes seemed to be the primary performance determining factor of visuomotor reaction speed while motor processes seemed to play only a minor role.

### Table Tennis vs. Badminton Athletes

Athletes participating in table tennis and badminton require extremely fast visuomotor reactions. Therefore, we compared the results of this study (*n* = 37) to our previous findings in badminton athletes (*n* = 36) and non-athletic control participants (*n* = 28) who participated in an identical reaction task for the 5 Hz velocity condition (Hülsdünker et al., 2017, 2018a). Interestingly, regarding the VMRT, badminton players (244.2 ms) only outperformed non-athletes (273.6 ms; *P* < 0.001) while the difference to table tennis athletes (258.4 ms) was not significant (*P* = 0.151). Further, although table tennis players were faster than non-athletes, this difference did not reach the significance threshold (*P* = 0.148). For the N2 potential, athletes from both sports exhibited a significantly lower latency (badminton: 182.5 ms; table tennis: 187.1 ms) when compared to non-athletes (199.7 ms; badminton: *P* < 0.001; table tennis: *P* = 0.012). Since the N2-r latency was higher in the two athlete groups (badminton: 26.30 ms; table tennis: 17.19 ms; non-athletes: −7.17 ms; *P* < 0.001) this suggests both badminton and table tennis players achieve a faster visual perception and processing speed when compared to non-athletes. Taken together, these findings support the suggestions that long-term training over many years induce adaptations in the visual motion system that facilitates the perception/processing of visual information and consequently accelerate visuomotor reactions. Importantly, the performance of the table tennis athletes is even more impressive given the difference in age when compared to the group of badminton players (Hülsdünker et al., 2018a) and non-athletes (Hülsdünker et al., 2017). Since both, the VMRT and N2 latency have been shown to accelerate during maturation until the age of around 20 (Fozard et al., 1994; Langrová et al., 2006) the faster VMRT in badminton players when compared to non-athletes may be attributable to age effects. Future studies investigating age-matched table-tennis and badminton athletes will provide more accurate results on performance differences between athletes in these two visuomotor demanding sports.

## Conclusion

This study is the first that identified the neural processes determining the visuomotor reaction speed of elite young table tennis athletes. We observed faster visuomotor reactions were predicted by the speed of visual information processing in the brain’s visual motion sensitive area MT while motor processes did not contribute to the visuomotor reaction performance. These findings and are well in line with previous results from highly-skilled adult badminton players. In sum, this study supports the importance of neural visual processes for visuomotor reactions and emphasize its generalizability across different visuomotor demanding sports and age groups.

## Data Availability

The raw data supporting the conclusions of this manuscript will be made available by the authors, without undue reservation, to any qualified researcher.

## Ethics Statement

This study was carried out in accordance with the recommendations of ethics committee of the German Sport University Cologne. All participants and parents were provided detailed study information several weeks prior to the experiment. Since subjects were under the legal age, parents/guardians gave their written informed consent to the study. All subjects gave written informed consent in accordance with the Declaration of Helsinki. The protocol was approved by the ethics committee of the German Sport University Cologne.

## Author Contributions

TH, MO and AM designed the experiment. TH and MO recruited subjects and conducted the experiments. TH and AM analyzed the data and drafted the manuscript. All authors reviewed and approved the final version of the manuscript. All authors agree to be accountable for all aspects of the work in ensuring that questions related to the accuracy or integrity of any part of the work are appropriately investigated and resolved.

## Conflict of Interest Statement

MO is employee of the FLTT and honorary co-worker of the CTTCE. Both institutions did not fund this work nor were they involved in any aspect of study design, data collection, data analysis and interpretation or writing and reviewing the manuscript.
